# A Single Oral Immunization with Replication-Competent Adenovirus-Vectored Vaccine Induces a Neutralizing Antibody Response in Mice against Canine Distemper Virus

**DOI:** 10.3390/v14091847

**Published:** 2022-08-23

**Authors:** Xiang Du, Emeline Goffin, Lucie Gillard, Bénédicte Machiels, Laurent Gillet

**Affiliations:** Laboratory of Immunology and Vaccinology, Faculty of Veterinary Medicine, FARAH, ULiège, 4000 Liège, Belgium

**Keywords:** oral vaccine, canine distemper virus, morbillivirus, replicative adenovirus vector

## Abstract

Canine Distemper Virus (CDV) is a fatal and highly contagious pathogen of multiple carnivores. While injectable vaccines are very effective in protecting domestic animals, their use in the wild is unrealistic. Alternative vaccines are therefore needed. Adenovirus (AdV) vectors are popular vaccine vectors due to their capacity to elicit potent humoral and cellular immune responses against the antigens they carry. In parallel, vaccines based on live human AdV-4 and -7 have been used in U.S. army for several decades as replicative oral vaccines against respiratory infection with the same viruses. Based on these observations, the use of oral administration of replication competent AdV-vectored vaccines has emerged as a promising tool especially for wildlife vaccination. Developing this type of vaccine is not easy, however, given the high host specificity of AdVs and their very low replication in non-target species. To overcome this problem, the feasibility of this approach was tested using mouse adenovirus 1 (MAV-1) in mice as vaccine vectors. First, different vaccine vectors expressing the entire or part H or F proteins of CDV were constructed. These different strains were then used as oral vaccines in BALB/c mice and the immune response to CDV was evaluated. Only the strain expressing the full length CDV H protein generated a detectable and neutralizing immune response to CDV. Secondly, using this strain, we were able to show that although this type of vaccine is sensitive to pre-existing immunity to the vector, a second oral administration of the same vaccine is able to boost the immune response against CDV. Overall, this study demonstrates the feasibility of using replicating AdVs as oral vaccine vectors to immunize against CDV in wildlife carnivores.

## 1. Introduction

Distemper is a fatal and highly contagious disease of young carnivores that results from infection with Canine Distemper Virus (CDV), a member of the genus Morbillivirus in the Paramyxoviridae family [1]. CDV is a large (100–250 nm), enveloped virus containing a 15,690 nt long unsegmented negative-stranded RNA genome consisting of six genes that encode a single envelope-associated protein [matrix (M)], two glycoproteins [the hemagglutinin (H) and fusion (F) proteins], two transcriptase-associated proteins [phosphoprotein (P) and large protein (L)] and the nucleocapsid (N) protein, which encapsulates the viral RNA [1]. Only one serotype of CDV is recognized, with several co-circulating genotypes based on variation in the H protein [2]. CDV is readily transmitted between susceptible hosts through contact with various body secretions containing the pathogen, including respiratory, oral and ocular fluids and urine. CDV enters a new host through the nasal or oral route, coming into contact with the epithelium of the upper respiratory tract [1]. The virus multiplies in tissue macrophages and then spreads to the tonsils and respiratory lymph nodes within 24 h, and to other lymphoid tissues within 2 to 4 days, resulting in severe immunodepression [3]. After one week, CDV reaches the gastrointestinal mucosa and the liver, resulting in a systemic reaction characterized by fever and leukopenia. Finally, other epithelial cells and the central nervous system are infected [4]. Clinical signs include fever, mucopurulent discharge from the eyes and the nose, cough, dyspnoea, depression, anorexia, vomiting and diarrhoea. In parallel, neurological signs occur within 2–3 weeks. They are progressive and vary depending on the area of the brain affected. Infection of the central nervous system results in the death of infected animals 2–4 weeks after infection [1].

Although CDV was originally described as a pathogen of domestic dogs, it has become a threat for multiple wild carnivore species worldwide [5,6]. For example, CDV infects canids (such as foxes, wolves, dingos and coyotes), felids (such as lions, tigers and leopards), procyonids (raccoon), ursids (bears), mustelids (ferrets, minks), hyenids and others. In particular, during the last three decades, several epidemics involving endangered species have been described worldwide and have, for example, devastated the Serengeti lion population [7]. Recently, fatal CDV infections have been observed in wild endangered species such as Amur tigers [8] and giant pandas [9]. Massive distemper outbreaks have also been reported in marine mammals, including seals, in which mass mortality has been linked to viral strains likely originating from land carnivores [10]. Finally, the ability of CDV to cross species barrier has been illustrated by its ability to infect non-human primates such as rhesus monkey and cynomolgus macaques with high mortality rates [6,11,12] raising several concerns about a potential zoonotic risk of CDV infection in humans [5,13,14].

Vaccine-based prophylaxis is likely the best effective way to keep clinical distemper disease under control [15]. Conventional live modified vaccines are commercially available and are widely used in dogs. These vaccines provide a rapid and robust immunity; however, their efficacy is limited in the presence of maternal-derived antibodies. The world small animal veterinary association considers the CDV vaccine as one of the three core vaccines for dogs, i.e., a vaccine against a severe, life-threatening diseases that all dogs should receive regardless of circumstances or geographical location [16]. While injection of CDV vaccines is highly efficient in domestic dogs, such an approach is unfeasible for wildlife. Alternative vaccination strategies are therefore needed. In particular, oral immunization may offer several advantages over the traditional parentally administrated vaccines and has previously revealed successful for wildlife populations. Indeed, oral rabies vaccination has resulted in virtual elimination of fox-mediated rabies from large parts of Western and Central Europe [17,18]. However, oral administration of live modified vaccines or of recombinant CDV canarypox-vectored vaccines [19,20] does not confer protection against distemper. Oral live adenovirus (AdV) vectors have many advantages for the development of oral vaccines. Accordingly, we have recently shown that mouse AdV type 1 (MAV-1) oral administration in mice induces a protective immune response against a homologous challenge [21] but can also be used as a viral vector to protect against heterologous infections such as influenza virus infection. In the present study, we exploited this mouse model to investigate the potential of replication-competent AdVs as vectors for oral vaccination against CDV.

## 2. Materials and Methods

**Animals.** All animal work complied with relevant European, federal, and institutional policies. The Committee on the Ethics of Animal Experiments of ULiège reviewed and approved the protocol (permit number 1526). Female BALB/c mice were purchased from Charles Rivers Laboratories, France. All mice were housed in ULiège, Department of Infectious Diseases. The animals were infected orally with MAV-1 when 7–8 weeks old. Briefly, viruses were diluted in 200 µL of Dulbecco’s Modified Eagle Medium (DMEM; Life Technologies, Carlsbad, CA, USA) and administered into the oesophagus with a steel feeding gavage needle under restraint of the mouse.

**Virus, cells and sera.** For this work we used the pmE101 wild type strain of MAV-1 [22,23] and *Escherichia coli* SW102 containing the pKBS2.MAV-1 wild type (WT) bacmid coding for the entire MAV-1 WT [24]. The CDV Onderstepoort strain expressing green fluorescent protein [25] (CDV-OndeGFP) was kindly provided by Prof. V. von Messling (Paul-Ehrlich-Institute, Langen, Germany), and the CDV-OR12 was kindly provided by Dr. B. Hu (Chinese Academy of Agricultural Sciences, Changchun, China). MAV-1 and CDV were grown on mouse 3T6 cells and Vero cells respectively, cultivated in DMEM, supplemented with 2 mM glutamine, 100 U penicillin mL^−1^, 100 mg streptomycin mL^−1^ and 5% heat-inactivated foetal calf serum (FCS) at 37 °C in an atmosphere of 5% CO_2_. Rabbit sera anti CDV F and H ectodomains (anti-F and anti-H) [26] were provided by Prof. V. von Messling (Paul-Ehrlich-Institute, Langen, Germany).

**Construction of MAV-1 recombinant vectors.** Total RNA was extracted from CDV infected Vero cells with trizol reagent (Thermo Fisher Scientific) and cDNA of H and F ORFs was amplified with H (Fwd: TCTCGCTTGATTGCCAGGTT and Rev: ACAGAGTCCATGGCTGAAAGG) or F (Fwd: ACAGGAACCCCCACAAACAG and Rev: AACCTGGCAATCAAGCGAGA) specific primers using PrimeScript RT-PCR Kit (Takara Bio, Kusatsu, Japan). Amplification products were ligated into pGEM-T Easy vectors (Promega, Madison, WI, USA) by TA cloning. To introduce CDV sequences into the MAV-1 genome, recombination arms at the 5′- and 3′-ends of CDV H and F genes were introduced by PCR using Phusion^®^ High-Fidelity PCR Master Mix (New England Biolabs, Ipswich, MA, USA) with primer pairs ArmH-FL (recombination arms are underlined and lowercase; the sequence of the H gene is uppercase Fwd: gaccttctcaagttggcaggagacgttgagtccaaccctgggcccATGCTCTCCTACCA and Rev: tttttattaaacataaagcgcgtgagcatgcatctttatttgggaTTAACGGTTACATGAG) and ArmF-FL (recombination arms are underlined and lowercase; the sequence of the F gene is uppercase Fwd: gaccttctcaagttggcaggagacgttgagtccaaccctgggcccATGCACAAGGGAATC and Rev: tttttattaaacataaagcgcgtgagcatgcatctttatttgggaTCAGAGTGATCTCACATAG) respectively. These cassettes (H-FL and F-FL) were cloned downstream and in frame with the MAV-1 pIX ORF. A sequence coding for a furin 2A cleavage site (agaaaaagaagggcaccggtgaaacagactttgaattttgaccttctcaagttggcaggagacgttgagtccaaccctgggccc) was added between pIX and H or F sequences to allow release of proteins as described [27].

Since the size of the transgenes is limiting for AdV vectors, we produced a short form of the F transgene in which the signal peptide sequence of CDV F was replaced by the shorter one of measles virus F gene using primers MeV-SP+F (measles signal peptide is double underlined Fwd: ATGGGTCTCAAGGTGAACGTCTCTGCCATATTCATGGCAGTACTGTTAACTCTCCAAACACCCACCGGTCAGATACATTGGAATAATTTGTC) and ArmMeV-SP (Measles virus F signal peptide is double underlined and uppercase; Rev: gaccttctcaagttggcaggagacgttgagtccaaccctgggcccATGGGTCTCAAGGTGA) to generate the recombination F-Short cassette.

Finally, the globular domain of CDV H protein (HG) was amplified from positions 1350 to 1815 of the CDV H ORF, which refers to amino acids 156-604 of the protein. The HG protein was expressed as either a non-structural (HG-NS) or structural protein (HG-S) of MAV-1 virions by introducing either the F2A cleavage site as described above or a protein linker [28] (GSAGSAAGSGEF) between pIX and the HG transgene. Briefly, the HG-NS cassette was produced by PCR with primers Arm HG-NS (recombination arms is underlined and lowercase; sequence of H gene is uppercase Fwd:gaccttctcaagttggcaggagacgttgagtccaaccctgggcccTCAATTGGGATCAG) and ArmH-FL reverse primer. The HG-S cassette was produced by two rounds of PCR with primers Linker HG-Str (recombination arms are in italics and lowercase; linker is in bold and uppercase Fwd: *aaagtgat***GGATCCGCTGGCTCCGCTGCTGGTTCTGGCGAATTC**TCAATTGGGATCAG) and Arm Linker (recombination arms is in Italic and lowercase; Linker is in bold and uppercase Fwd: *aagaggaggacggagctgaagacattgaggaaaacggggaagaaagtgat***GGATCCGCT**) as successive forward primers and ArmH-FL as reverse primer.

Recombinants were produced using *Escherichia coli* SW102 containing the pKBS2.MAV-1 WT bacmid coding for the entire MAV-1 WT [24] and prokaryotic recombination technologies as described previously [29]. Briefly, a GalK cassette with a sequence encoding furin 2A cleavage site (agaaaaagaagggcaccggtgaaacagactttgaattttgaccttctcaagttggcaggagacgttgagtccaaccctgggccc) at 5′ was introduced in place the of pIX stop codon to allow positive selection of recombinants on a minimal medium, then the inserted Galk was replaced by the H-FL, F-FL, F-Short, HG-NS or HG-S cassettes allowing counter-selection on a DOG medium toxic for non-recombinants. The screening of recombinants was made by PCR and confirmed by restriction profile. After BAC purification, the mutated MAV-1 genome was excised from the plasmid by I-SceI (NEB, Ipswich, MA, USA) and transfected in 3T6 cells to rescue the recombinant viruses. To confirm the molecular structure of the recombinant strains, viral genomes were purified with QIAamp DNA Mini Kit (Qiagen, Hilden, Germany) and PCR encompassing the insertion site were performed on the viral genome of the different recombinant strains with primers check pIX Fwd: TCTCGGCTGTTGCACGAAGAT and check pIX Rev CGTCAGCGACAAAGGTGGAAC.

**Restriction profile.** BAC containing MAV-1 or recombinants were extracted from overnight cultured SW102 *E. coli*. Viral genomic DNA was extracted from purified virions as previously described [30]. DNA (2 µg) was digested with *Apa*I for 2 h at 25 °C. The digestion products were separated by electrophoresis on a 0.8% agarose gel.

**Indirect immunofluorescence staining of adherent cells.** 3T6 cells plated on coverslip were either mock-infected or infected with the different MAV-1 recombination strains. Cells 72 h p.i. were washed once in PBS, fixed in 4% paraformaldehyde for 10 min at room temperature and washed twice in PBS. Cells were either non-permeabilized or permeabilized in PBS 0.1% NP-40 for 15 min at room temperature. Unspecific binding was blocked in PBS with 10% foetal calf serum (FCS) before incubated with primary antibodies. Cells were then incubated overnight at 4 °C with rabbit sera directed against CDV F or H proteins ectodomains (anti-Fect and anti-Hect) [26] diluted 800-fold and with mouse sera directed against MAV-1 diluted 1000-fold. After washing, goat anti-rabbit-Alexa 488 and goat anti-mouse-Alexa 568 antibodies (Invitrogen, Waltham, MA, USA) were applied at a 1:1000 dilution for 30 min at room temperature before three washes in PBS. The nucleus was stained by 4′, 6-diamidino-2-phenylindole (DAPi; Merck Group, Darmstadt, Germany). The cover slips were washed in PBS for 5 min and rinsed once with distilled water, dried and then mounted with ProLong Gold Antifade Mountant (ThermoFisher, Geel, Belgium) on glass slides. Z-stack acquisition images were captured on Leica SP5 confocal microscope and image files were processed with ImageJ.

**Growth curve.** Viral growth of recombinants was characterized in the 3T6 cell line. Briefly, 5 × 10^4^ cells were plated in 24-well plates and infected at 500 TCID50. Virus titers were then determined at days 0, 3, 6, 9, 12 and 15 post infection. Briefly, cells and supernatants were submitted to triple freeze-thaw cycles to release cellular virion before centrifugation to remove cellular debris. Supernatants were then used to infect 2.66 × 10^5^ 3T6 cells in 12-well plates in 2% FCS DMEM (Final volume fixed to 2 mL). Seventy-two post infection, cells were harvested in Cell Dissociation Buffer (Thermo Fisher Scientific, Waltham, MA, USA), fixed in 4% paraformaldehyde for 20 min then blocked in 10% FCS PBS for 30 min containing anti-mouse CD16/32 antibody (BioLegend, San Diego, CA, USA). Mouse anti-MAV1 sera was then used at a 1:800 dilution in 10% FCS PBS blocking buffer for 1h under shaking. After washing in PBS, anti-Mouse-Alexa Fluor 488 (Invitrogen; 1:800) was applied for 15 min. All the incubations were performed on ice and followed by a PBS wash. For each sample, 10,000 cells were registered on a BD FACSAria III.

**Quantification of MAV-1 and CDV specific antibodies by in-cell ELISA.** 3T6 cells were plated in 96-well plates at 1 × 10^4^ cells per well, infected with 2500 TCID_50_ of MAV-1 per well, and incubated at 37 °C, 5% CO_2_ for 5 days. Vero cells were plated in 96-well plates at 2 × 10^4^ cells per well, infected with 2500 TCID_50_ of CDV per well, and incubated at 37 °C, 5% CO_2_ for 2 days. Then, cells were fixed in 2% paraformaldehyde for 20 min at RT, washed two times in PBS and blocked for 30 min with PBS, 10% FCS, 300 mM glycine. Cells were then incubated at 4 °C for 2 h with mouse sera diluted 100-fold in the blocking solution. Cells were washed three times in PBS and incubated at room temperature for 30 min with a goat anti-mouse Ig polyclonal secondary antibody conjugated with alkaline phosphatase (0.5 µg/mL, Sigma, Darmstadt, Germany). After three washes with PBS, p-nitrophenyl phosphate (Sigma) was added and incubated for one hour, then the reaction was stopped with 1 M sodium hydroxide. Absorbance was measured at 405 nm using a benchmark ELISA plate reader (Thermo). Signals detected from mock infected cells were used as background and this background was subtracted from the corresponding values obtained with MAV-1 or CDV infected wells.

**Neutralization Assays.** A plaque microneutralization assay was carried out. Briefly, Vero cells were seeded the day before the experiment at 10^4^ cells per well of a 96-well plate. Serial two-fold dilution of the heat-inactivated sera (56 °C for 30 min) was mixed with an equal volume of 100 TCID50 CDV-OndeGFP in DMEM to reach final dilutions of 1/32 to 1/1024 in 100 µL. The serum–virus mixture was incubated for 2 h at 37 °C and added to the cells. After an absorption period of 2 h, cells were supplemented with 100 µL of DMEM 4% FCS. Control wells (i.e., virus without serum) were averaged to represent 100% relative infection. 48 h later, the infection was detected by expression of GFP to read neutralizing titres which was considered as the highest dilution of serum without GFP expression.

**Statistical analysis.** Data were analysed with GraphPad Prism 5.0 software. For all the experiments, *n* = 5 in each group. The data were analysed by Wilcoxon–Mann–Whitney rank-sum test.

## 3. Results

### 3.1. Design and Generation of MAV-1 Recombinants Expressing CDV Antigens

**Figure 1:**
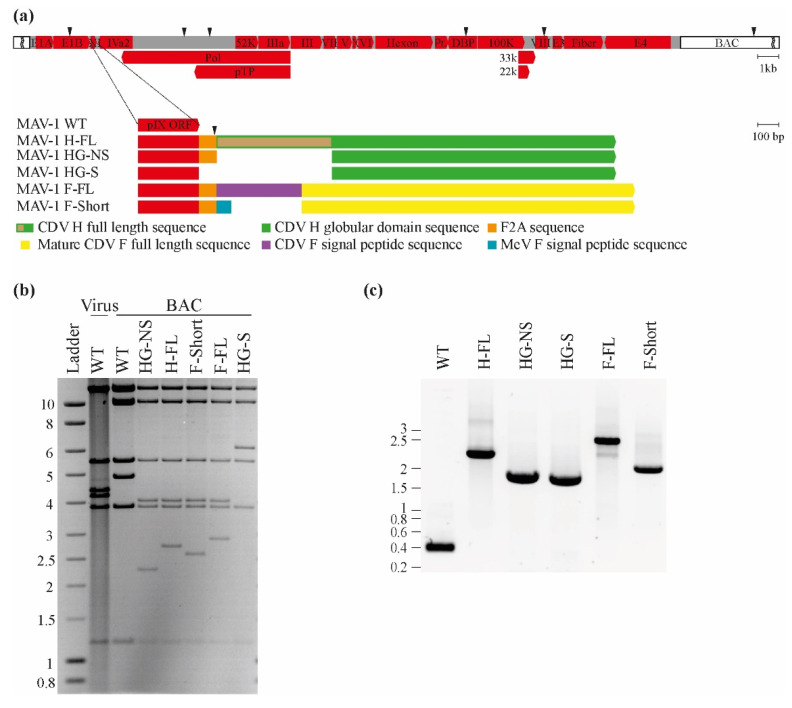
Construction and characterization of MAV-1 recombinant expressing CDV antigens. (**a**) Schematic representation of the strategies used to produce the recombinant MAV-1 strains. The MAV-1 genome is represented from 5′ to 3′ in a Bacterial Artificial Chromosome (BAC). Black arrowheads indicate *Apa*I restriction sites. (**b**) Verification of the molecular structures. Viral DNA and BAC DNA were isolated and digested with *Apa*I. The restriction fragments were then separated by electrophoretic migration and revealed by ethidium bromide and UV exposure. Pr.: protease. Marker sizes in Kbp are indicated on the left. (**c**) PCR amplification of the region encompassing the insertion site. The PCR products were separated by electrophoretic migration and revealed by ethidium bromide and UV exposure. Marker sizes in Kbp are indicated on the left.

We constructed MAV-1 recombinant vectors expressing either the full-length H or F proteins of CDV Onderstepoort strain or shortened version of them. AdV vectors have a packaging capacity limitation of about 105% of the length of their wild-type genomic DNA [31]. The full-length sequences of CDV F and H proteins correspond to 6.43% and 5.87% of MAV-1 genome length, respectively, and could potentially impede virion production. Therefore, beside insertion of full length ORFs, we tried to reduce the DNA size of the transgenes. First, we designed a chimeric F gene in which the CDV F signal peptide was replaced by the equivalent and much shorter sequence of measles virus, reducing DNA insertion to 5.34% of the MAV-1 genome. In parallel, as the globular domain of the CDV H protein is immunodominant [32], we only expressed this region in some mutants, reducing the insertion size to 4.36% of the MAV-1 genome length. All of these transgenes were inserted downstream of the MAV-1 protein IX (pIX) sequence in which the pIX stop codon was replaced by a furin 2A cleavage sequence linking transgenes expression to transcription of pIX without addition of transcriptional control regions [27] (Figure 1a). The resulting mRNA allows giving rise to distinct proteins through enzymatic cleavage of the furin 2A site. Finally, in one recombinant expressing the H globular domain, we directly fused the transgene sequence to the sequence of pIX in order to express this transgene as a structural component of MAV-1 virions. Altogether, we generated five MAV-1 recombinant strains encoding either a non-structural H (H-FL), a non-structural H globular domain (HG-NS), a structural H globular domain (HG-S), a non-structural F (F-FL) and a non-structural shorter version of F (F-short) (Figure 1). The constructions of these MAV-1 recombinants were checked by *Apa*I enzymatic restrictions of the BAC genomes, showing the expected profiles (Figure 1b) and confirmed by sequencing of recombination regions performed on viral genomes (data not shown). Moreover, to exclude any genome rearrangement, PCR encompassing the recombination regions were performed to assess the size of the inserted transgenes. The results obtained showed that only one band was amplified at the expected size (WT: 399 bp, H-FL: 2298 bp, HG-NS: 1833 bp, HG-S: 1749 bp, F-FL: 2472 bp and F-Short: 2136 bp) confirming that all the vectors have the correct molecular structure (Figure 1c).

**Figure 2:**
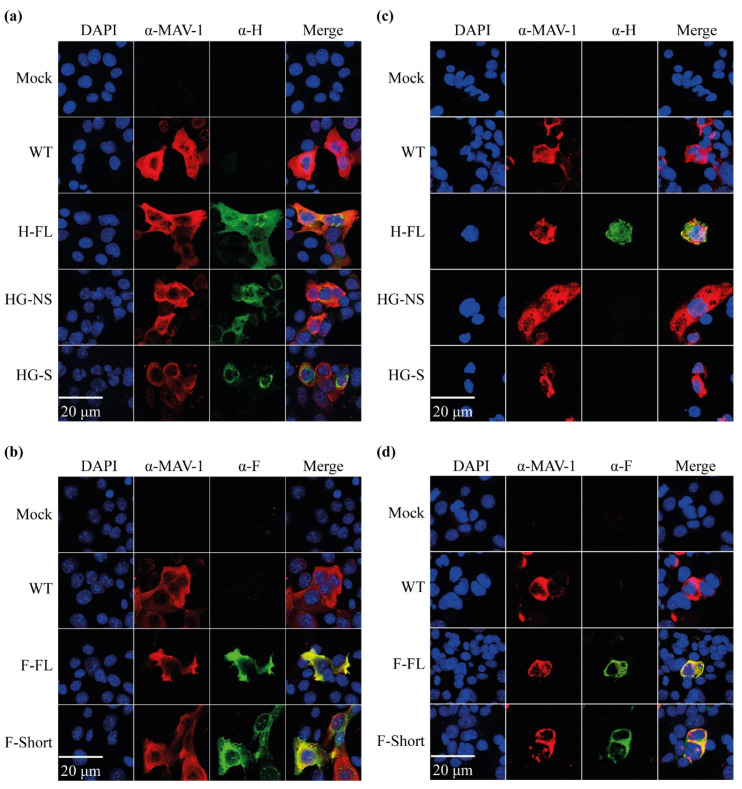
Validation of MAV-1 mediated expression of CDV antigens in vitro. The different MAV-1 strains were grown on 3T6 cells for 72 h (MOI of 0.01). Cells were then fixed and permeabilized (panels (**a**,**b**)) or not (panels (**c**,**d**)). Fluorescent immunolabeling was performed using mouse polyclonal antibodies against MAV-1 detected by Alexa 568 goat anti-mouse polyserum. CDV antigens were labeled with rabbit anti-H ectodomain (panels (**a**,**c**)) and anti-F ectodomain (panels (**b**,**d**)) detected by Alexa 488 goat anti-rabbit polyserum. Cells nuclei were visualized with DAPI.

In order to check the expression of the transgenes, 3T6 cells were infected with the different MAV-1 recombinants and submitted to immunofluorescence for MAV-1 and CDV antigen detection after permeabilization or not. We firstly analysed expression of CDV H protein. In permeabilized cells, signals were observed from H-FL, HG-NS and HG-S infected cells stained with a rabbit polyserum raised against CDV H protein ectodomain (Figure 2a). In contrast, in non-permeabilized cells, only H-FL-infected cells displayed an immunofluorescent signal (Figure 2c), confirming that H-FL expression is distributed at the surface of infected cells while the HG-NS and HG-S forms are retained internally. The CDV F protein is naturally located at the cell surface as well [33]. Accordingly, F expression from the two different MAV-1 recombinant strains was detected both in permeabilized and non-permeabilized cells (Figure 2b,d). Interestingly, we did not identify a significant difference in F expression between the two MAV-1 recombinant strains. These results show that all recombinants expressed the CDV antigens as expected.

**Figure 3:**
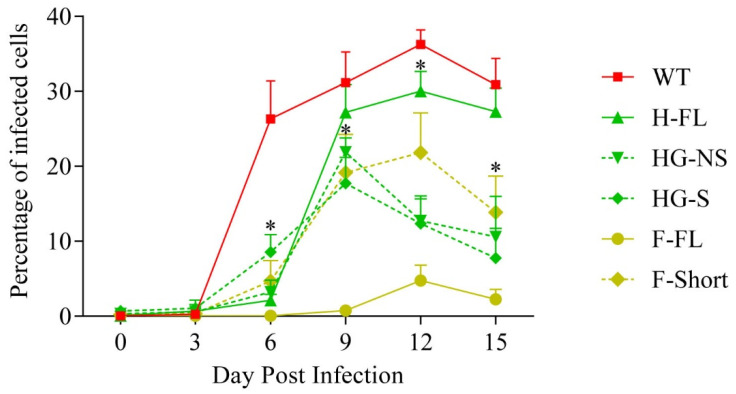
Growth of MAV-1 recombinants. 3T6 cells were infected with 500 TCID50 of the different MAV-1 strains and harvested at the indicated time points to titer virus production as described in the methods. The data presented are means for triplicated measurements ± SEM. *p* values are relative to comparison with the growth of MAV-1 WT strain. *, *p* < 0.05.

Multi-step growth curves were carried out on 3T6 cells to compare the growth of the recombinant viruses with that of WT MAV-1 (Figure 3). This revealed that all recombinant strains displayed reduced growth in comparison with WT. In particular, the F-FL strain displayed the largest defect, which is in accordance with the size of the foreign CDV material inserted into its genome. Interestingly, while the H-FL strain has a longer genome than the HG-NS, HG-S and F-short, it displayed a lower growth deficit in comparison with WT. Accordingly, in comparison with the MAV-1 WT strain, the titers that could be reached for the production of the vectors were up to 2 log lower for the H-FL recombinant, 3 log lower for HG-NS, HG-S and F-short and 4 log lower for the H-FL strain. Altogether, these results suggest that all MAV-1 recombinants have a growth deficit in comparison with the WT strain, even if this defect seems smaller for the MAV-1 H-FL strain.

### 3.2. Oral Immunization with the MAV-1 H-FL Strain Induces Anti-CDV Immune Response

**Figure 4:**
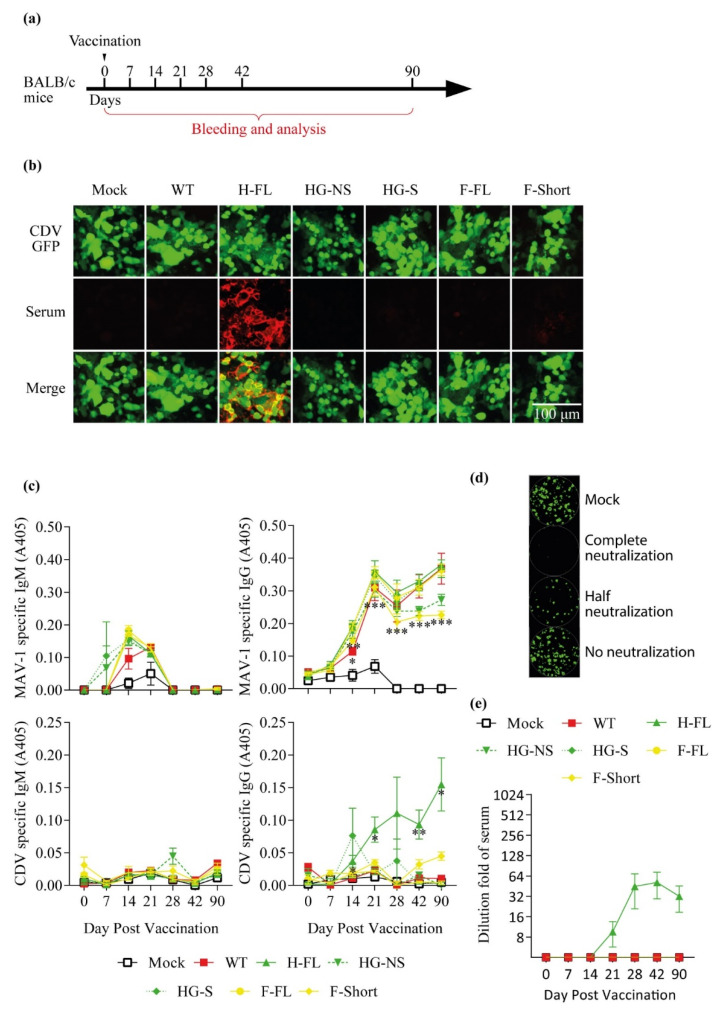
Characterization of the immune response induced by the MAV-1-based oral vaccines in BALB/c mice. (**a**) Design of the experiment. 8-week-old female BALB/c mice (*n* = 5 mice/group) were orally immunized with 600 TCID50 of WT, H-FL, HG-NS, HG-S, F-FL or F-Short MAV-1 strains or with PBS as control (mock). Blood samples were collected at the indicated times p.i. (**b**) Detection of anti-CDV seroconversion by immunofluorescence performed on CDV-OndeGFP infected Vero cells with mice sera taken at 28 days post vaccination. Mouse antibodies were detected with Alexa 568 goat anti-mouse polyserum. (**c**) MAV-1 and CDV specific IgG and Ig measured over time in mouse serum as described in the methods. The data presented are the means for 5 mice ± SEM. *p* values are relative to comparison with the mock group. *, *p* < 0.05, **, *p* < 0.01, ***, *p* < 0.001. (**d**) Representative images of the neutralizing effect. The mock condition represents treatment with serum of non-vaccinated mice. (**e**) CDV-neutralizing antibody titres measured over time in mouse serum as described in the methods. The data presented are the means for 5 mice ± SEM.

To evaluate the ability of orally administered AdV vectored vaccines to confer an immune response against CDV, mice were immunized by gavage with 600 TCID50 of the different MAV-1 recombinants or, as negative controls, the same dose of MAV-1 WT or PBS only (mock) (Figure 4a). Mice were observed and weighed every other day from the day before vaccination until day 9 post-immunization. None of the mice developed any clinical signs after vaccination (data not shown). Similar to the protective situation in measles, the presence of neutralizing antibodies against CDV, acquired through previous vaccination or exposure to field virus, predicts protection against the disease [34,35,36,37]. Serum was collected at the indicated times post-infection, then MAV-1 and CDV specific (Figure 4b,c) and neutralizing (Figure 4d) antibodies were measured by immunofluorescence, in-cell ELISA and virus neutralization assay, respectively. Oral immunization with all strains induced a transient IgM and a sustained IgG response against MAV-1 (Figure 4c). In contrast, only immunization with MAV-1 H-FL gave rise to significant levels of specific (Figure 4b,c) and neutralizing (Figure 4d) antibodies against CDV.

### 3.3. Impact of Pre-Existing Immunity against MAV-1 on the Capacity of MAV-1 H-FL Strain to Induce Anti-CDV Immune Response

**Figure 5:**
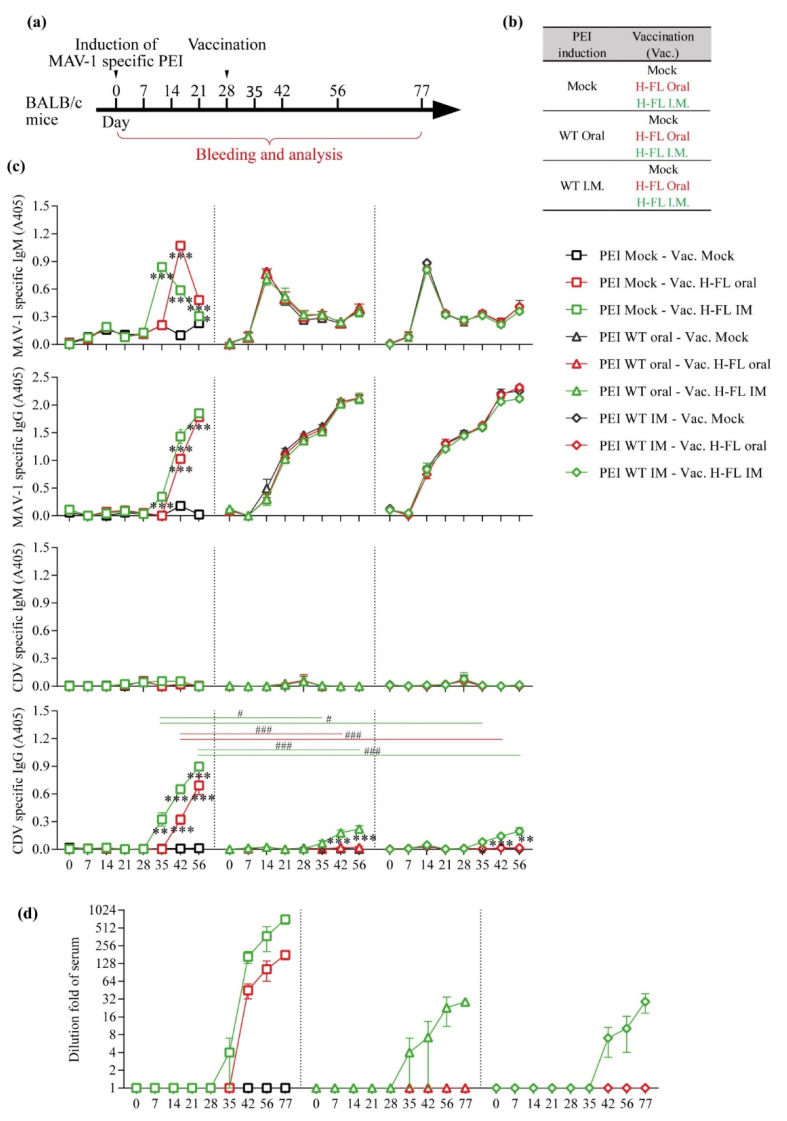
Effect of pre-existing immunity on primary vaccination efficiency. (**a**,**b**) Design of the experiment. Anti-MAV-1 specific pre-existing immunity was induced in 8-week-old female BALB/c mice (*n* = 5 mice/group) through oral or intramuscular infection with 2000 TCID50 of MAV-1 WT as described in the methods. Mice were then immunized, or not, with 2000 TCID50 of the H-FL MAV-1 strain at day 28 via the oral or the intramuscular route. Blood samples were taken at the indicated time points. (**c**) MAV-1 and CDV specific IgG and IgM measured over time in mouse serum as described in the methods. The different routes to induce pre-existing immunity are represented by symbols (mock: □; oral: △; IM: ◇) and the different routes of vaccination with the H-FL strain of MAV-1 are labeled by colors (mock: black; oral: red; I.M.: green). The data presented are the means for 5 mice ± SEM. *p* values represented with stars (*) are relative to comparison with the Vac. mock group. *, *p* < 0.05, **, *p* < 0.01, ***, *p* < 0.001. *p* values represented with hashtags (#) are relative to comparison with the PEI mock group. #, *p* < 0.05, ###, *p* < 0.001. (**d**) CDV-neutralizing antibody titres measured over time in mouse serum as described in the methods. The symbols and color code are identical those of (**c**). The data presented are the means for 5 mice ± SEM.

Pre-existing immunity to AdV is a major impediment to the induction of an immune response against transgenes expressed by AdV-based vector vaccines [38]. However, studies have suggested that this is not the case when these vaccines are administered orally [39]. To test whether pre-existing immunity to MAV-1 would affect immunization to CDV H protein with the MAV-1 H-FL strain, we administered the WT strain of MAV-1 orally or intramuscularly to groups of 5 BALB/c mice to induce pre-existing immunity. On day 28 post-infection, these mice were then vaccinated or not with the MAV-1 H-FL strain administered orally or intramuscularly. Blood samples were taken at the indicated time points and the serological response to the MAV-1 vector and to CDV was monitored (Figure 5a,b). Interestingly, we did not observe any difference in the timing of the appearance of IgM or IgG against MAV-1 between the groups infected orally or intramuscularly with the WT strain (Figure 5c). However, in the uninfected and subsequently vaccinated groups, IgM against the MAV-1 vector appeared approximately 1 week later in the orally vaccinated animals compared to the intramuscularly vaccinated ones (Figure 5c). In the same groups, a similar delay was observed in the detection of IgG against CDV. Unexpectedly, pre-existing immunity to MAV-1 strongly reduced the ability of the vaccine to induce an immune response to CDV H protein regardless of the route of administration used (Figure 5c). However, it should be noted that while no anti-CDV serological response could be detected in orally vaccinated animals, a reduced response was detected after intramuscular immunisation in the presence of pre-existing anti-MAV-1 antibodies (Figure 5c) suggesting that intramuscular administration is less affected than the oral adminstration. Serum neutralization experiments confirmed this result (Figure 5d). These results thus show that pre-existing immunity to MAV-1 blocks the ability of MAV-1-based vaccines to induce immunity to the transgene, particularly when administered orally, compared to the intramuscular route.

### 3.4. Pre-Existing Immunity Does Not Block the Ability of a Second Administration of the Oral Vaccine to Boost Immunity against the Transgene

**Figure 6:**
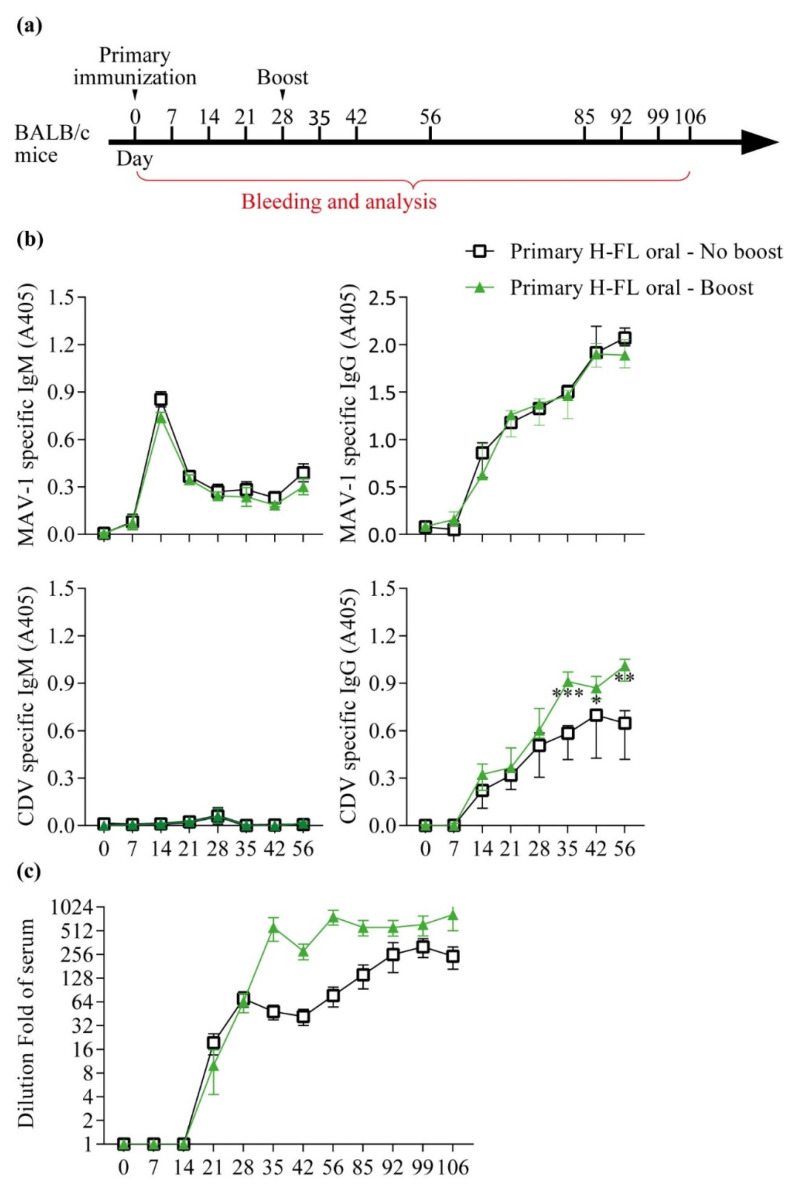
Boosting immune response with oral vaccination. (**a**) Design of the experiment. Eight-week-old female BALB/c mice (*n* = 5 mice/group) were orally immunized with 2000 TCID50 of H-FL MAV-1 strain or with PBS as control (mock). Mice were then immunized, or not, with 2000 TCID50 of the H-FL MAV-1 strain at day 28 via the oral route. Blood samples were collected at the indicated times p.i. (**b**) MAV-1 and CDV specific IgG and IgM measured over time in mouse serum as described in the methods. The data presented are the means for 5 mice ± SEM. *p* values are relative to comparison with the mock group. *, *p* < 0.05, **, *p* < 0.01, ***, *p* < 0.001. (**c**) CDV-neutralizing antibody titres measured over time in mouse serum as described in the methods. The symbols and color code are identical to those in (**b**). The data presented are the means for 5 mice ± SEM.

The results obtained above call into question the validity of strategies based on two administrations of the same vaccine by the oral route. To test whether this is the case or not, two groups of BALB/c mice were vaccinated using the MAV-1 H-FL strain. Twenty-eight days after the first administration of the vaccine, one of the two groups received a second oral administration of the same vaccine. Blood samples were taken from the animals at different times after the primary vaccination (Figure 6a). Surprisingly, while the IgM or IgG response against the MAV-1 vector was not affected, a second administration made it possible to significantly increase the quantity of anti-CDV antibodies (Figure 6b) and the ability of serum to neutralize this virus (Figure 6c). These results suggest that despite pre-existing immunity, it may be advantageous to administer twice the same vaccine based on replicative AdV vectors in order to boost the immune response against a transgene.

## 4. Discussion

Distemper, caused by CDV, is a devastating disease for many species of domestic and wild carnivores. In addition, many recent events of transmission to species that are a priori non-susceptible, including non-human primates, raise fears of the possible emergence of new zoonotic diseases from this virus [32]. Control of CDV in domestic and wild animal populations is therefore a crucial objective for both veterinary and human public health. Vaccination is a strategy of choice for combating the CDV virus by the induction of neutralizing antibodies against the two surface glycoproteins of the virus, proteins F and H [32]. In domestic animals, for many years, vaccination strategies based on attenuated vaccine strains have been successful, and several international associations of veterinary practitioners consider vaccination against CDV to be an essential vaccination that all dogs should receive at least once in their lifetime [16]. At the level of wild carnivore species, the situation is more complicated, given that it is not possible to use injectable vaccines in these animals. Strategies based on oral vaccines are therefore very promising alternatives. Recently, the use of replicative AdV vectors has been proposed in the context of the development of oral vaccination [40]. For wild carnivores, canine adenovirus 2 (CAV-2) is certainly a vector of choice in this context [41,42]. However, the species barrier obliges us to use a murine homolog, the MAV-1 virus, to develop this type of strategy in a preclinical model easily usable in the laboratory, i.e., the mouse. In this study, based on the MAV-1 virus used as an oral replicative vaccine vector, we constructed different vaccine strains expressing vaccine antigens derived from the H and F proteins of the CDV virus (Figure 1) and tested their potential in vitro and in vivo.

We first showed, in vitro, that the CDV antigens can be expressed within cells infected by these recombinant MAV-1 strains and present an intracellular or cell surface localization as expected (Figure 2). One of the major problems of AdV vectors, in particular replicative ones, is their ability to insert foreign genetic material. It is commonly accepted to consider that these vectors can incorporate genetic material of a size of that can reach up to 105% of the size of the initial viral genome [31]. In the context of replicative vectors, this capacity for insertion is very limited, given that most of the viral genes must be conserved. In the context of this study, we chose to use an insertion site downstream of the pIX gene for the following two reasons. First, this insertion site had been described before [43]. Second, the nature of the junction zone makes it possible to express the transgene either as an isolated protein after cleavage by cellular furin 2A, or as a fusion protein with the pIX protein, and thus to be expressed on the surface of the viral particle. Interestingly, viral strains expressing all the transgenes tested could be obtained, thus confirming the quality of the chosen insertion zone. All these recombinant strains, however, displayed a reduction in their replicative capacity (Figure 3). Surprisingly, this reduction in growth was not proportional to the size of the transgene used, since the strain expressing the entire H protein replicated better than the strains expressing only the globular part of this same protein (Figure 3). In the case of the F protein, we were nevertheless able to show that the use signal peptide coding sequence of the measles virus F protein made it possible to reduce the size of the inserted transgene in comparison with a transgene composed of the gene of the F protein of CDV, and was accompanied by better growth even if it was still strongly reduced in comparison with the WT strain of MAV-1 (Figure 3). Finally, an interesting piece of information is that the strains expressing the globular part of the H protein in the form of non-structural or structural protein presented similar growths (Figure 3), indicating that this type of expression is feasible.

With regard to the immunogenic power of these different vaccine strains, we were only able to detect a serological response and a neutralizing response against the CDV virus in the context of the use of the MAV-1 strain expressing the whole protein H (Figure 4). Previous studies had already demonstrated the quality of this transgene as a vaccine antigen [44]. It should be noted, however, that in our framework this recombinant strain was the one which showed the best growth (Figure 3). It is therefore quite difficult to distinguish the effect resulting from the nature of the transgene from that resulting from the growth properties of the recombinant strain, especially since these two aspects are most certainly linked. Since this study was dedicated to establishing a proof of concept for oral vaccination against CDV, we then continued with this single strain expressing the entire H protein.

We only assessed the ability of these recombinant vaccines to induce a humoral response against CDV. Indeed, similar to the immune response against measles virus, neutralizing antibodies are sufficient to confer protection against distemper disease [34,35,36,37]. In the future, as adenoviral vectors are known to induce a good cellular immune response, it will be interesting to use the approach described in this study in a context where this type of response is essential.

A major question related to the use of AdV as vaccine vectors lies in the influence that a pre-existing immunity against the vector itself may have on the ability of the vaccine to induce an immune response against the transgene. Very interestingly, several studies, carried out in a mouse model, have suggested that the oral administration of the vaccine could make it possible to escape this pre-existing immune response [39]. However, our observations do not support this conclusion in the context of vaccines based on replicative AdV. Indeed, in animals with pre-existing immunity, the MAV-1 H FL strain induced a less good immune response against the transgene after oral administration than after intramuscular administration (Figure 5). This greater sensitivity of oral administration under our experimental conditions may have several origins. In particular, the vaccination scheme in this study is based on the administration of a relatively small quantity of infectious particles, given the replicative nature of the vaccine which allows amplification of the expression of the transgene. The need for this amplification could therefore make this approach much more sensitive to pre-existing immunity against the vector than approaches based on non-replicating or poorly replicating vectors. This greater sensitivity could be particularly revealed by the oral route, given the probably very low number of initially infected cells. Intramuscular administration may be less affected because vaccine particles are likely to maintain better infectivity within muscle than within the gut lumen. Therefore, the number of infected cells could be much higher after intramuscular administration than after oral immunization. Inactivated or non-replicating vaccines, which is the case for the vaccines previously studied in this context, could result in a different conclusion given that no amplification step is necessary in their case. This study therefore suggests that in addition to the route of immunization, the type of vaccine determines its sensitivity to pre-existing immunity against the vector.

The sensitivity to pre-existing immunity raises questions, particularly in the context of immunization protocols requiring the administration of two vaccine doses, or in the context of the use of the same vector for different vaccines. Nevertheless, the results obtained within the framework of this study show that a boost of the immune response is possible via the administration of a second dose of the same vaccine (Figure 6). Furthermore, the long-term persistence of this interference by pre-existing immunity against the vector will be worth testing in the future. This question is particularly important in view of the widespread use of AdV vector-based vaccines in the context of SARS-CoV-2 control, even if these are not replicative vectors [45]. In order to limit problems related to pre-existing immunity, these vaccines have mainly been developed on the basis of AdV of infrequent serotypes, or from other animal species such as the chimpanzee. However, recent studies tend to show that pre-existing immunity to these vectors does not influence immunisation to the transgene [46].

Finally, although feasible, the use of replicative AdV vectors should be the subject of a benefit/risk study. Indeed, it has been shown that these vaccine vectors can be disseminated through the body after oral administration [21]; therefore, the safe use of these replicative vaccines should be investigated on a large scale, and more deeply in animal models such as the one used here.

Taken together, the results obtained in this study show the feasibility of developing a vaccine against CDV based on replicative AdV vectors. They show, however, that these oral vaccines are likely to be more sensitive to the presence of pre-existing immunity against the vector than the same vaccines administered intramuscularly, although a second administration of these vaccines by the same oral route can boost the immune response against the transgene.
